# The complete plastome of *Centaurium erythraea* subsp. *majus* (Hoffmanns. & Link) M.Laínz (Gentianaceae), the first chloroplast genome belonging to the *Centaurium* genus

**DOI:** 10.1080/23802359.2022.2160670

**Published:** 2023-01-09

**Authors:** Inês Carvalho Leonardo, Adriana Alberti, France Denoeud, Maria Teresa Barreto Crespo, Jorge Capelo, Frédéric Bustos Gaspar

**Affiliations:** a iBET, Instituto de Biologia Experimental e Tecnológica, Oeiras, Portugal; b ITQB-NOVA, Instituto de Tecnologia Química e Biológica António Xavier, Universidade Nova de Lisboa, Oeiras, Portugal; c Génomique Métabolique, Genoscope, Institut François Jacob, CEA, CNRS, Univ Évry, Université Paris-Saclay, Évry, France; d ECOCHANGE, CIBIO-InBIO – Research Centre in Biodiversity and Genetic Resources, Universidade do Porto, Vairão, Portugal; e INIAV, Instituto Nacional de Investigação Agrária e Veterinária I.P., Quinta do Marquês, Oeiras, Portugal

**Keywords:** *Centaurium erythraea* subsp. *majus*, Gentianaceae, complete chloroplast genome, Illumina MiSeq sequencing, phylogenetic analysis

## Abstract

Despite having many historically reported ethnomedicinal uses, *Centaurium erythraea* Rafn (Rafn and Buchs, 1800; common centaury) also produces cytotoxic secondary metabolites, and its presence should be carefully monitored. In this study, the complete chloroplast of *Centaurium erythraea* subsp. *majus* (Hoffmanns. & Link) M.Laínz (Laínz, 1971) isolate BPTPS121 is described, being the first available plastome belonging to the *Centaurium* genus. The chloroplast genome (GenBank accession number: ON641347) is 153,107 bp in length with 37.9% GC content, displaying a quadripartite structure that contains a pair of inverted repeat regions (25,166 bp each), separated by a large single-copy (84,388 bp) and small single-copy (18,387 bp) regions. A total of 129 genes were predicted, including 37 tRNA genes, eight rRNA genes, and 84 protein-coding genes. The phylogenetic analysis showed that isolate BPTPS121 is placed under the Gentianaceae family, belonging to the Gentianales order. The maximum-likelihood tree supports the already described lineage divergence in the Gentianaceae family, with *C. erythraea* subsp. *majus* belonging to the Chironieae tribe positioned below the Exaceae tribe and above the Potalieae and the entire Gentianeae tribes. This study will contribute to conservation, phylogenetic, and evolutionary studies, as well as DNA barcoding applications for food, feed, and supplements safety purposes.

*Centaurium* Hill (Hill et al. 1756), a genus of flowering plants in the Gentianaceae family commonly known as ‘centauries’, has included ca. 108 species in the past. This previously polyphyletic genus has since been reclassified and redistributed among different genera. For example, ca. 25 species of New World centauries were transferred to the *Zeltnera* G.Mans. genus, ca. five species to *Gyrandra* Griseb. (Mexico and Central America), and ca. five to *Schenkia* Griseb. (Australia and Pacific) (Mansion 2004). This redistribution has left *Centaurium* sensu stricto with ca. 20 species (Struwe 2014), distributed mainly around the Western Mediterranean region while reaching the Balkan Peninsula.

*Centaurium erythraea* Rafn (Rafn and Buchs 1800; common centaury) is the most abundant species of which many ethnomedicinal uses have been historically reported. Significantly, it is one of the plants described and depicted in the work De Materia Medica by the celebrated Greek medical writer Dioscorides (40–90 BCE). Common putative uses have been described as anti-bacterial, anti-fungal, anti-leishmanial, insecticidal, anti-oxidant, anti-inflammatory, anti-diabetic, and anti-proliferative, as well as gastroprotective, hepatoprotective, dermoprotective, and neuroprotective, among others (El Menyiy et al. 2021). These various pharmacological properties arise from the production of several classes of secondary metabolites, namely xanthonoids, terpenoids, flavonoids, phenolic acids, and fatty acids. From these, significant antibacterial activity has been attributed to two secoiridoid glycosides, swertiamarin and sweroside, as well as cytotoxicity (Kumarasamy et al. 2003) and, as such, their presence should be carefully monitored.

The material of *Centaurium erythraea* subsp. *majus* (Hoffmanns. & Link) M.Laínz (Laínz 1971) analyzed (BioSample: SAMN28118559; Figure 1) was collected from a wild population in Torres Vedras municipality (Dois Portos) in Portugal (collection date: 2020-06-15; location: 39.06612 N, 9.1788 W). This plant material was identified as isolate BPTPS121 with a specimen being conserved at the LISE Herbarium (INIAV, Oeiras, Portugal; Jorge Capelo: jorge.capelo@iniav.pt) under the voucher LISE: 96442 (identified by: Jorge Capelo).

**Figure 1.**
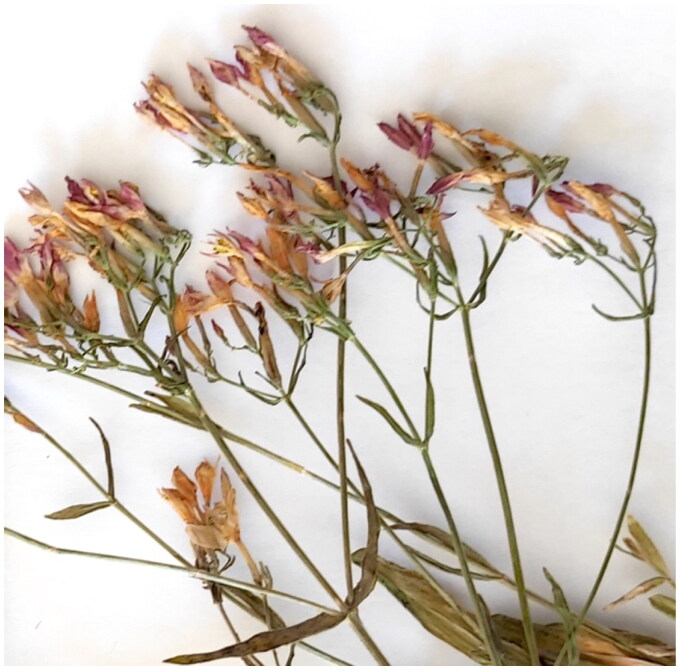
Detail of *C. erythraea* subsp. *majus* isolate BPTPS121 (BioSample: SAMN28118559) after being dried and before being mounted and conserved at the LISE Herbarium (INIAV, Oeiras, Portugal; Jorge Capelo: jorge.capelo@iniav.pt) under the voucher LISE: 96442 (identified by: Jorge Capelo). This isolate was collected from a wild population in Torres Vedras municipality (Dois Portos) in Portugal (collection date: 15 June 2020; location: 39.06612 N, 9.1788 W).

Immediately after collection, young leaves from the selected specimen were frozen in liquid nitrogen and stored at −80 °C until further processing. Total genomic DNA was extracted from the preserved material using an adaptation of the Doyle and Doyle (1987) methodology. After quantity (Qubit 4 Fluorometer, Thermo Fisher Scientific, Waltham, MA) and quality (NanoDrop ND-1000, Thermo Fisher Scientific, Waltham, MA) evaluation, the obtained DNA was sent to Genoscope (Évry, France) for sequencing. DNA was first sonicated using the Covaris E210 Focused Ultrasonicator instrument (Woburn, MA), and then libraries were prepared with the NEBNext Ultra II DNA Library Prep Kit (New England Biolabs, Ipswich, MA). Finally, sequencing was performed using 151 base-length read chemistry in a paired-end flow cell on the Illumina NovaSeq 6000 sequencing platform (San Diego, CA).

The about 40 million high-quality paired-end reads obtained (SRA: ERR10047930) were used to assemble the complete chloroplast genome (sequence coverage: 972×) using the GetOrganelle pipeline (v1.7.3.1) (Jin et al. 2020). The pipeline was used following the typical recipe suggested for Embryophyta plant plastome assembly (https://github.com/Kinggerm/GetOrganelle) while setting the flags ‘--max-reads’ and ‘--reduce-reads-for-coverage’ to 25 million and one thousand, respectively (see Supplemental material for additional details). The plastome annotation was performed using the GeSeq tool (Tillich et al. 2017) using the default parameters and the provided 3rd party stand-alone annotators Chloë (v0.1.0). A subsequent manual curation of the obtained annotations was performed using Geneious Prime 2022.0.1 (https://www.geneious.com) to compare them with the results obtained by performing a BLAT search on GeSeq (protein, rRNA, tRNA, DNA search identities set to 90%; see Supplemental material for additional details).

The chloroplast genome of *C. erythraea* subsp. *majus* isolate BPTPS121 (GenBank accession number: ON641347; Figure 2) is 153,107 bp in length with 37.9% GC content, displaying a quadripartite structure that contains a pair of inverted repeat (IR) regions (25,166 bp; GC content 43.4%), separated by a large single-copy (LSC) region (84,388 bp; GC content 36.0%) and a small single-copy (SSC) region (18,387 bp; GC content 31.7%). A total of 129 genes were predicted (113 of them unique), including 37 tRNA genes (30 of them unique), eight rRNA genes (four of them unique), and 84 protein-coding genes (79 of them unique).

**Figure 2.**
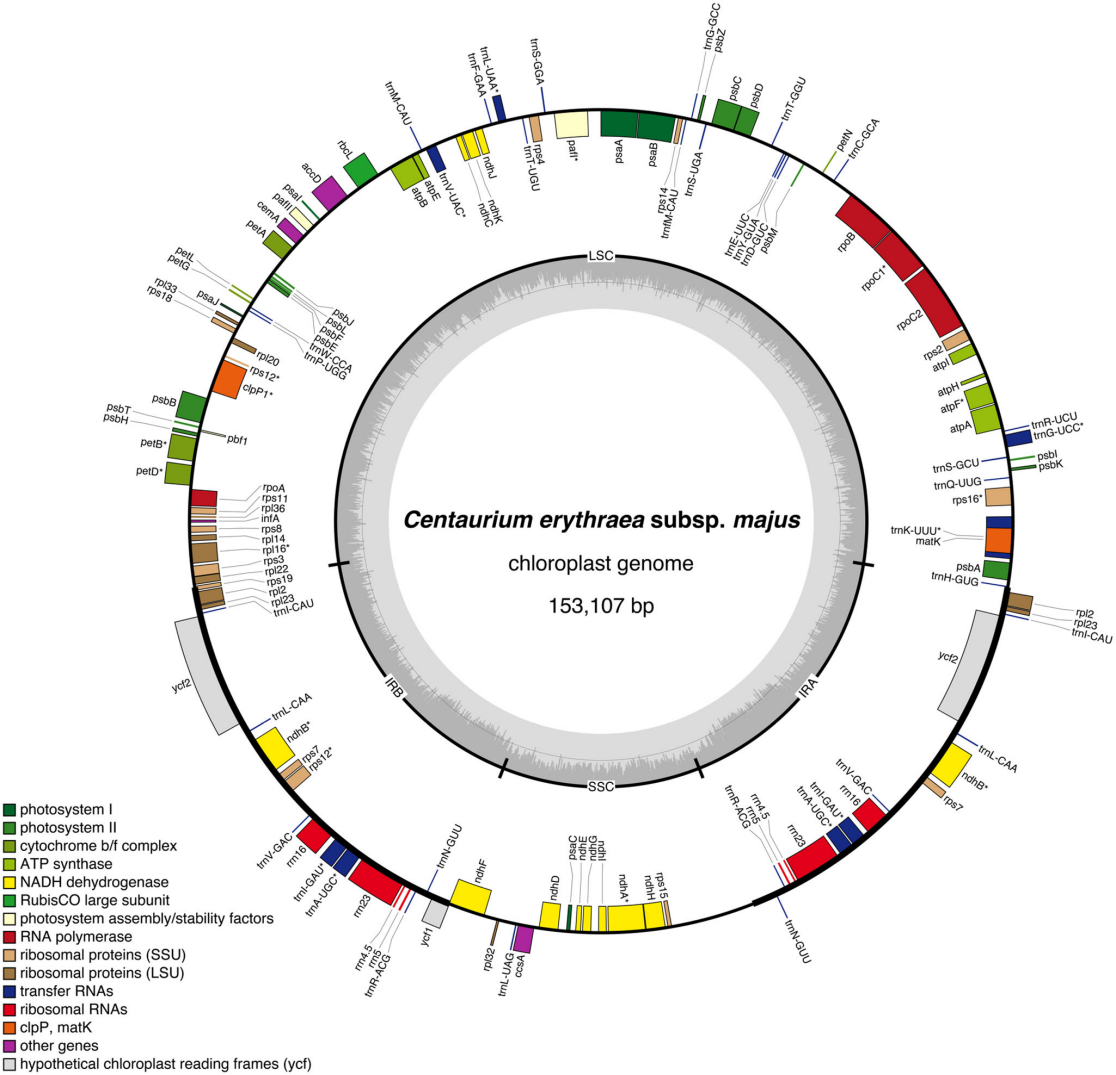
Graphical map of the complete chloroplast genome of *Centaurium erythraea* subsp. *majus* isolate BPTPS121 based on the conversion of annotations openly available in GenBank (accession number: ON641347), color coded based on their functional group, using OrganellarGenomeDRAW (OGDRAW) version 1.3.1 (Greiner et al. 2019). Genes inside the circle are transcribed clockwise, genes outside the circle counterclockwise, and intron-containing genes are marked by an asterisk (*). LSC: large single-copy region; SSC: small single-copy region; IRA, IRB: inverted repeats (IR). The dark grey inner ring represents the GC content, while the complementary light grey ring represents the AT content.

The phylogenetic analysis (see Supplemental material for additional details) was performed using the concatenated nucleotide sequences coding for the shared proteome (65 coding sequences) extracted from a selected dataset. The dataset was composed of 17 verified and complete chloroplast genomes belonging to the Gentianaceae family available in GenBank (accession date: 26 June 2022), with only one species representing each genus. The selected dataset also included the complete chloroplast genome of *C. erythraea* subsp. *majus* isolate BPTPS121 obtained in this study and three additional sequences used as outgroups in the phylogenetic analysis: *Catharanthus roseus* (L.) G.Don (NC_021423; Apocynaceae family) belonging to the Gentianales order but not from the Gentianaceae family, *Nonea vesicaria* (L.) Rchb. (NC_060826; Boraginales order) belonging to the same lamiids clade but from a different order, and *Tilia platyphyllos* Scop. (NC_062378; Malvales order) from the malvids clade. The sequences were aligned using MAFFT v7.450 (Katoh and Standley 2013) and further analyzed with the IQ-TREE 2 software package (Minh et al. 2020). The best-fit substitution model (TVM + F+I + I+R2 chosen according to the Bayesian information criterion) was selected according to ModelFinder (Kalyaanamoorthy et al. 2017), followed by a tree reconstruction (Figure 3) using IQ-TREE (Nguyen et al. 2015) using ultrafast bootstrap with UFBoot (10,000 replicates) (Hoang et al. 2018).

**Figure 3.**
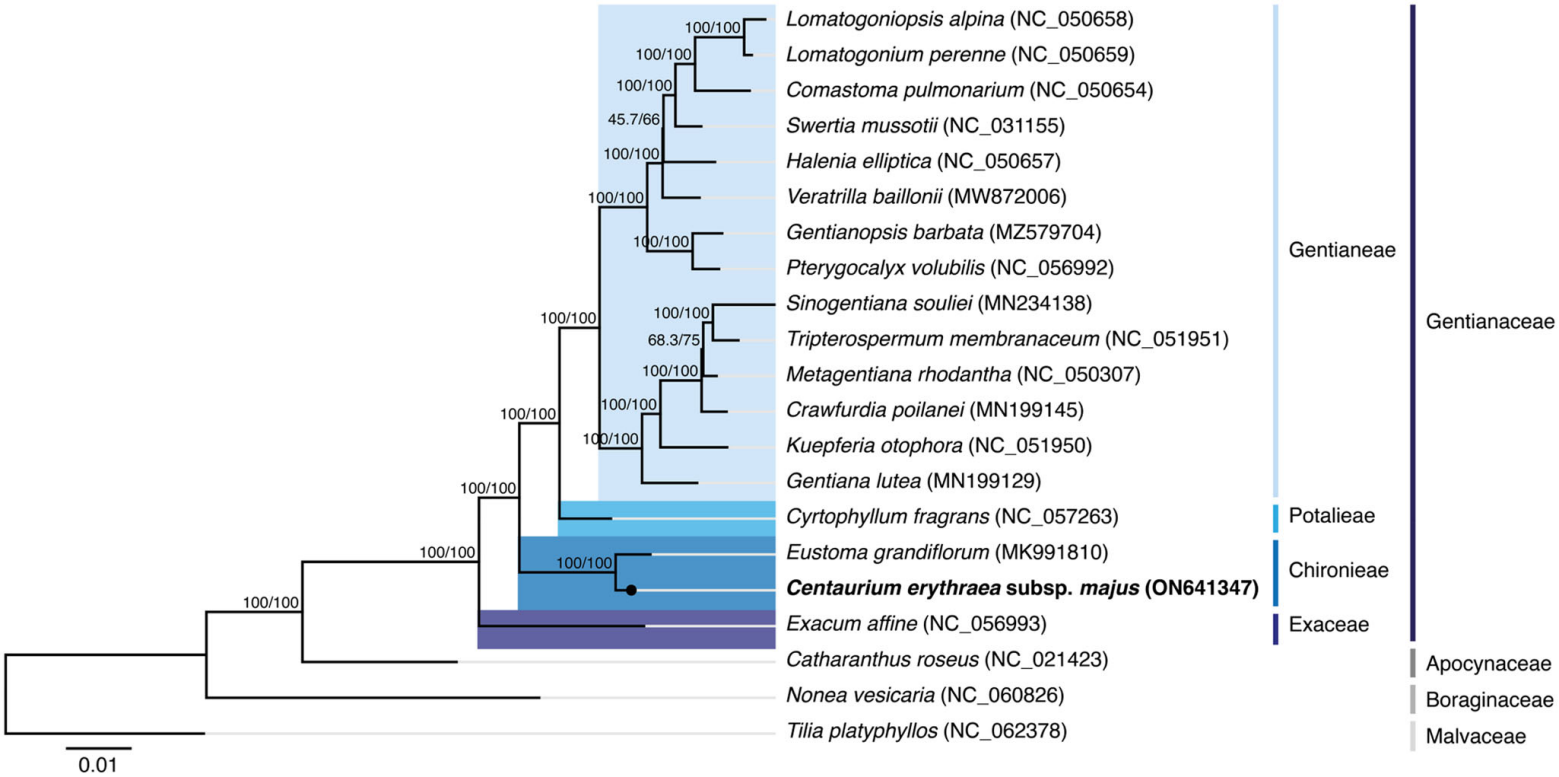
Maximum-likelihood tree inferred from the sequences coding for the shared proteome from *Centaurium erythraea* subsp. *majus* isolate BPTPS121 and 17 verified and complete chloroplast genomes belonging to the Gentianaceae family available in GenBank (accession date: 26 June 2022). The numbers attached to the branches show the SH-aLRT and the UFBoot2 percent supports (SH-aLRT/UFBoot2). *Catharanthus roseus* (L.) G.Don (NC_021423; lamiids clade, Apocynaceae family), *Nonea vesicaria* (L.) Rchb. (NC_060826; lamiids clade, Boraginales order), and *Tilia platyphyllos* Scop. (NC_062378; malvids clade, Malvales order) were used as the outgroups.

The maximum-likelihood tree showed that *C. erythraea* subsp. *majus* isolate BPTPS121 is placed under the Gentianaceae family, belonging to the Gentianales order. The Gentianaceae family has six tribes in the current classification: the Chironieae, Exaceae, Gentianeae, Helieae, Saccifolieae, and Potalieae (Struwe 2014). These tribes are unevenly represented in GenBank’s genome resources, with Gentianeae having 163 unique, verified, and complete chloroplast genomes, Chironieae, Exaceae, and Potalieae with one unique entry each, and Helieae and Saccifolieae with none. Using the available data, the phylogenetic analysis performed supports that the Chironieae tribe (*C. erythraea* subsp. *majus* isolate BPTPS121 (ON641347) and *Eustoma grandiflorum* (MK991810)) is the second most basally positioned tribe, below Exaceae (*Exacum affine*; NC_056993), and above Potalieae (*Cyrtophyllum fragrans*; NC_057263) and the entire Gentianeae tribe, with 100/100 percent support (SH-aLRT/UFBoot2). This tree, therefore, supports the already described lineage divergence in the Gentianaceae family (Struwe 2014), with complete chloroplast genomes of isolates from the Helieae and Saccifolieae tribes still missing in the databases, as well as for the still uncertain placement of *Voyria*. The phylogenetic analysis performed using the concatenated amino acid sequences of the shared proteomes also supports the same phylogenetic result (see Supplemental material for additional details).

This study describes the chloroplast genome of *C. erythraea* subsp. *majus* isolate BPTPS121, the first described plastome belonging to the *Centaurium* genus. This complete genome will contribute to conservation, phylogenetic, and evolutionary studies in the Gentianaceae family. It will also support DNA barcoding applications for food, feed, and supplements safety and quality purposes that target detecting species that produce secondary metabolites with cytotoxic potential.

## Data Availability

The data that support this study is openly available in GenBank of NCBI at https://www.ncbi.nlm.nih.gov under the accession number ON641347. The associated BioProject, BioSample, and SRA numbers are PRJNA848681, SAMN28118559, and ERR10047930, respectively.
